# Genomic Analysis of *LEA* Genes in *Carica papaya* and Insight into Lineage-Specific Family Evolution in Brassicales

**DOI:** 10.3390/life12091453

**Published:** 2022-09-19

**Authors:** Zhi Zou, Jingyuan Guo, Yujiao Zheng, Yanhua Xiao, Anping Guo

**Affiliations:** Hainan Key Laboratory for Biosafety Monitoring and Molecular Breeding in Off-Season Reproduction Regions, Institute of Tropical Biosciences and Biotechnology, Sanya Research Institute of Chinese Academy of Tropical Agricultural Sciences, Haikou 571101, China

**Keywords:** papaya (*Carica papaya*), brassicales, late embryogenesis abundant protein, orthogroup, abiotic stress, expression profile

## Abstract

Late embryogenesis abundant (LEA) proteins comprise a diverse superfamily involved in plant development and stress responses. This study presents a first genome-wide analysis of *LEA* genes in papaya (*Carica papaya* L., Caricaceae), an economically important tree fruit crop widely cultivated in the tropics and subtropics. A total of 28 members were identified from the papaya genome, which belong to eight families with defined Pfam domains, i.e., LEA_1 (3), LEA_2 (4), LEA_3 (5), LEA_4 (5), LEA_5 (2), LEA_6 (2), DHN (4), and SMP (3). The family numbers are comparable to those present in *Ricinus communis* (Euphorbiaceae, 28) and *Moringa oleifera* (Moringaceae, 29), but relatively less than that found in *Moringa oleifera* (Cleomaceae, 39) and *Arabidopsis thaliana* (Brassicaceae, 51), implying lineage-specific evolution in Brassicales. Indeed, best-reciprocal-hit-based sequence comparison and synteny analysis revealed the presence of 29 orthogroups, and significant gene expansion in *Tarenaya* and *Arabidopsis* was mainly contributed by whole-genome duplications that occurred sometime after their split with the papaya. Though a role of transposed duplication was also observed, tandem duplication was shown to be a key contributor in gene expansion of most species examined. Further comparative analyses of exon-intron structures and protein motifs supported fast evolution of this special superfamily, especially in *Arabidopsis*. Transcriptional profiling revealed diverse expression patterns of *CpLEA* genes over various tissues and different stages of developmental fruit. Moreover, the transcript level of most genes appeared to be significantly regulated by drought, cold, and salt stresses, corresponding to the presence of *cis*-acting elements associated with stress response in their promoter regions. These findings not only improve our knowledge on lineage-specific family evolution in Brassicales, but also provide valuable information for further functional analysis of *LEA* genes in papaya.

## 1. Introduction

Late embryogenesis abundant (LEA) proteins comprise a large and diverse superfamily that is widely involved in plant development as well as stress responses [1,2,3]. Since their first discovery as accumulating late in cotton (*Gossypium hirsutum*) embryogenesis [4,5,6], over the past four decades, LEA proteins have been found in a wide range of plants as well as bacteria, fungi, and animals [1,7]. According to sequence similarity and particular Pfam domains present, LEAs can be classified into eight main families, i.e., LEA_1 (Pfam accession number PF03760), LEA_2 (PF03168), LEA_3 (PF03242), LEA_4 (PF02987), LEA_5 (PF00477), LEA_6 (PF10714), DHN (dehydrin, PF00257), and SMP (seed maturation protein, PF04927) [3,8,9]. In the model plant arabidopsis (*Arabidopsis thaliana*), the presence of 51 LEA-encoding genes was reported, whereby two members (i.e., AtEM10 and AtEM17) comprise one more family named AtM without significant protein domains [2,10]. Generally, LEA proteins are extremely hydrophilic; however, some members in the LEA_2 family were shown to be hydrophobic and even have a three-dimensional structure [11]. Increasing evidence shows that the accumulation of LEA proteins is not only found in seeds, but also different vegetative tissues especially under stress conditions, e.g., high temperature, low temperature, drought, and salt [2,3,12,13]. Moreover, improved stress tolerance was also observed after overexpressing *LEA* genes in *Escherichia coli*, yeast (*Saccharomyces cerevisiae*), and several model plants such as tobacco (*Nicotiana tabacum*), arabidopsis, and rice (*Oryza sativa*) [14,15,16,17]. Although the exact mechanism has not been clarified, LEA proteins are able to stabilize other proteins and membrane structures during water stress [16,18].

Papaya (*Carica papaya* L., *2n* = 18) is an important tree fruit crop that belongs to the Caricaceae family within the order Brassicales, which also includes arabidopsis as a representative in Brassicaceae, spider flower (*Tarenaya hassleriana*) in Cleomaceae, and horseradish tree (*Moringa oleifera*) in Moringaceae. Compared with the occurrence of two recent whole-genome duplications (WGDs) in both spider flower and arabidopsis, papaya and horseradish tree did not experience any additional WGD after the ancient so-called γ WGD shared by all core eudicots [19,20,21,22]. Although originated in Central America, the high nutritional value with significant vitamins and minerals in papaya fruits has prompted its wide cultivation in tropics and subtropics, e.g., India, Nigeria, Brazil, Mexico, Indonesia, and China [23]. In contrast to the considerable drought tolerance of wild relatives, commercial papaya cultivars are highly susceptible to cold and drought stresses [24,25], which frequently occur in subtropical regions such as south China. Therefore, exploring genes involved in stress responses and breeding resistant varieties in these areas are of particular importance. By taking advantage of available genome and transcriptome datasets, in this study, we would like to report a genome-wide analysis of *LEA* genes in papaya, which includes gene locations, exon-intron structures, sequence characteristics, evolutionary relationships, and *cis*-acting elements in the promoter regions, as well as gene expression patterns with a focus on fruit development and stress responses. These findings provide a global view of *CpLEA* genes that can facilitate further functional studies, and the comparative analysis with arabidopsis, spider flower, horseradish tree, and castor bean (*Ricinus communis*) contributes to our knowledge on the lineage-specific evolution of this special superfamily in Brassicales.

## 2. Materials and Methods

### 2.1. Data Retrieval and Identification of LEA Genes in Papaya, Horseradish Tree, and Spider Flower

*LEA* genes reported in arabidopsis and castor bean (see Table S1) were retrieved from Araport11 (https://www.arabidopsis.org/, accessed on 18 June 2022) and Phytozome v13 (https://phytozome-next.jgi.doe.gov/, accessed on 18 June 2022), respectively. Their protein sequences were used to identify homologs from papaya, horseradish tree, and spider flower, whose genome sequences were accessed from Phytozome v13, NCBI (http://www.ncbi.nlm.nih.gov/, accessed on 18 June 2022), and NGDC (https://ngdc.cncb.ac.cn/, accessed on 18 June 2022). The *E*-value of the tBLASTn search [26] was set to 1 × 10^−^^5^, and gene models of candidates were curated with available mRNAs as described before [27]. The presence of certain Pfam domains was confirmed using MOTIF Search (https://www.genome.jp/tools/motif/, accessed on 18 June 2022). Systematic names were assigned with two italic letters denoting the source organism and family name followed by a progressive number of their locations on chromosomes (Chrs) or scaffolds (Scfs).

### 2.2. Synteny Analysis and Gene Expansion Patterns

Homolog pairs were identified using the all-to-all BLASTP method (*E*-value cutoff 1 × 10^−^^10^) and syntenic blocks were inferred using MCScanX (BLAST hits ≥ 5) [26,28]. Tandem repeats were defined when two paralogs were consecutive in a genome; WGD repeats were considered when duplicated genes were located in syntenic blocks of duplicated chromosomes, and transposed repeats were identified using the DupGen_finder pipeline as previously described [29]. Orthologs between different species were determined using the Best Reciprocal Hit (BRH) method [30], as well as information from synteny analysis; and orthogroups (OGs) were assigned only when they were present in at least two species examined.

### 2.3. Exon-Intron Structure, Phylogenetic Analysis, and Structural Characterization

The exon-intron structure was analyzed using GSDS 2.0 [31] by aligning the coding sequence (CDS) to the corresponding genomic sequence. The molecular weight (MW), theoretical isoelectric point (pI), and grand average of hydropathy (GRAVY) were calculated using ProtParam (http://web.expasy.org/protparam/, accessed on 18 June 2022), and protein subcellular localization was predicted using WoLF PSORT (http://www.genscript.com/wolf-psort.html, accessed on 18 June 2022). Multiple sequence alignment and phylogenetic reconstruction were performed using MEGA6 [32] with MUSCLE and the maximum likelihood method (bootstrap: 1000 replicates), respectively. Conserved motifs in LEA proteins were identified using MEME (v 5.4.1) [33]: any number of repetitions; maximum number of motifs, 20; minimum sites, 2; and, the optimum width of each motif, between 6 and 100 residues.

### 2.4. Promoter Analysis

PLACE (http://www.dna.affrc.go.jp/PLACE/, accessed on 18 June 2022) was used to examine the presence of two stress-related *cis*-acting elements (i.e., abscisic acid response (ABRE, ACGTG) and low temperature response (LTRE, CCGAC)) in the 2000-bp promoter region of *CpLEA* genes.

### 2.5. Plant Materials, RNA-seq, and Gene Expression Analysis

Gene expression profiles were analyzed on the basis of RNA sequencing (RNA-seq) samples as shown in Table S2. Various tissues, i.e., root, apical bud, leaf, petiole, leaf vein, male flower, female flower, fruit, peel, and seed, were collected from one-year-old hermaphrodite plants of the cultivar Zhongbai that were planted in 2019 at the Wenchang experimental base, Institute of Tropical Biosciences and Biotechnology, Chinese Academy of Tropical Agricultural Sciences (Wenchang, Hainan, China: 19°32′15.39″ N, 110°45′47.26″ E). Routine management was performed, and three groups of more than five trees were used. As for cold and salt stresses, eight-week-old plantlets were used and treatments of 4 °C low temperature (i.e., 0, 7, 21, and 40 h) and 300 mmol/L NaCl (i.e., 0, 10, 15, and 20 d) were applied. To ensure the consistency of materials, only the second leaf from the top of a plantlet was collected and at least 10 leaves were pooled for total RNA isolation and subsequent Illumina RNA-seq as previously described [34,35]. As for drought stress, watering was withheld from three-month-old plants for 0, 10, and 20 d; and samples of roots, leaves, and phloem sap were sequenced as previously described [36]. Quality control and read mapping were carried out using Trimmomatic [37] and TopHat (v2.0.8) [38], respectively. The gene expression level was represented using FKPM (fragments per kilobase of exon per million fragments mapped) [39], and differentially expressed genes were determined using RSEM (v1.2.27) [40] with default parameters.

## 3. Results

### 3.1. Identification, Chromosome Localization, and Synteny Analysis of 28 LEA Genes in Papaya

**Figure 1:**
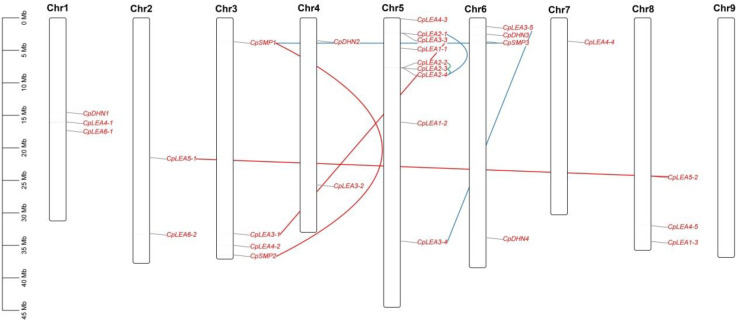
Chromosomal locations and duplication events of 28 *CpLEA* genes. Chromosome serial numbers are indicated at the top of each chromosome. *CpLEA2-4/-3/-2* are clustered as tandem repeats (lines in green); *CpLEA2-1/-4*, *CpLEA3-4/-5*, and *CpSMP1/3* are transposed repeats (lines in blue); and *CpLEA3-1*/*-3*, *CpLEA5-1*/*-2*, and *CpSMP1*/*2* are WGD repeats (lines in red) that are located in syntenic blocks.

Thus far, three genome assemblies have been reported in papaya, i.e., two for a virus-resistant transgenic variety SunUp, and one for its progenitor Sunset [20,41]. Whereas the ASGPBv0.4 assembly of SunUp is fragmented in 17,766 scaffolds [20], two recently available assemblies for SunUp and Sunset are chromosomal-level genomes [41], providing a good chance for comparative genomics analysis. Since the *LEA* genes identified in two chromosomal-level genomes are exactly the same, only results from the Sunset genome, as well as the ASGPBv0.4 assembly, were presented in Table 1, where an ortholog (i.e., sunset04G0003920/evm.TU.supercontig_6.122) of *AtLEA13/-43* was not included due to the absence of a significant LEA_4 domain. Based on the presence of Pfam domains in deduced proteins, 28 identified *CpLEA* genes were assigned into eight out of nine families as described in arabidopsis (only excluding the AtM family), and each family contains two to five members, respectively, i.e., *CpLEA1-1* to *-3*, *CpLEA2-1* to *-4*, *CpLEA3-1* to *-5*, *CpLEA4-1* to *-5*, *CpLEA5-1* to *-2*, *CpLEA6-1* to *-2*, *CpDHN1* to *-4*, and *CpSMP1* to *-3* (Table 1). Gene localization analysis indicated that they are not randomly distributed across eight out of nine chromosomes (excluding Chr9), varying from one (i.e., Chr7) to nine (i.e., Chr5) genes. Notably, several hotspots were observed, and a good example is the top of Chr5, which contains the maximum of seven genes (Figure 1). Correspondingly, eight duplicate pairs were identified, which include two tandem repeats (*CpLEA2-4/-3/-2*) and three transposed repeats (*CpLEA2-1/-4*, *CpLEA3-4/-5*, and *CpSMP1/-3*) (Table S1); on the contrary, synteny analysis revealed that the other three duplicate pairs are located in syntenic blocks and thus were defined as WGD repeats, i.e., *CpLEA3-1*/*-3*, *CpLEA5-1*/*-2*, and *CpSMP1*/*-2*. Among them, *CpLEA2-4/-3/-2/-1* as well as *CpLEA3-3* are located in the top region of Chr5, though *CpLEA3-4* is located in the bottom region (Figure 1). Whereas the protein identity between tandem repeats CpLEA2-3 and CpLEA2-4 is relatively low (about 29.0%), *CpLEA2-2* and *CpLEA2-3* exhibit 51.1% and 47.2% sequence identity at the nucleotide or protein level, respectively. Moreover, the first 483-bp sequences (counting from the initiation codon) of these two genes even harbor a relatively high sequence identity of 88.4%, and the low sequence identity of the full CDS was shown to result from the divergence of 3′ sequences (Figure S1).

### 3.2. Identification of LEA Genes in Horseradish Tree and Spider Flower and Definition of Orthogroups

**Figure 2:**
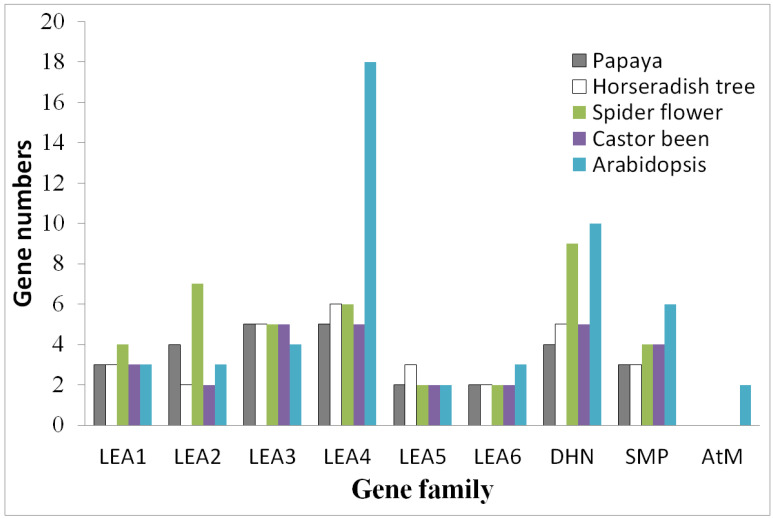
Distribution of papaya, horseradish tree, spider flower, arabidopsis, and castor been *LEA* genes in nine defined gene families.

The finding of almost half the amount of *LEA* genes in papaya relative to those in arabidopsis impelled us to investigate the lineage-specific evolution of the *LEA* superfamily in different families of Brassicales, i.e., Caricaceae, Moringaceae, Cleomaceae, and Brassicaceae. For this purpose, *LEA* genes were also identified from horseradish tree and spider flower, whose genome sequences have recently been accessible [21,22]. As shown in Table S1, 29 *LEA* genes identified in the horseradish tree are comparable to 28 present in papaya, as well as castor bean (an Euphorbiaceae plant also not having experienced any recent WGD), relatively less than 39 found in spider flower, and considerably less than 51 reported in arabidopsis, implying lineage-specific gene contraction and expansion. The species-specific distribution of *LEA* genes in nine defined gene families is summarized in Figure 2. Notably, no AtM homolog was found beyond arabidopsis.

To gain insights into species-specific evolution patterns, we further conducted BRH-based homology analysis between different species, resulting in 29 orthogroups that are present in more than one species compared (Table 2). In total, 28 *CpLEA* genes belong to 27 orthogroups, and each orthogroup includes one, with the exception of LEA2b containing two. As for two other orthogroups, DHNe is only present in horseradish tree and spider flower, whereas LEA4f is widely found, though a papaya homolog (see above) has lost the LEA_4 domain. Among three species without a recent WGD, i.e., papaya, horseradish tree, and castor bean, nearly one-to-one orthologous relationships were observed, though no member was identified in castor bean for LEA2b, DHNe, or LEA4a. Notably, a LEA4a homolog is actually found in castor bean, i.e., 30074.t000080; however, no significant LEA_4 domain was identified, supporting species-specific divergence. Like papaya, orthogroups that include more than one member were also found in horseradish tree and castor bean, i.e., MoLEA5-2/-3 in LEA5b, RcDHN2/-3 in DHNb, and RcSMP1/-2 in SMPb, all of which were characterized as tandem repeats (Table S1). On the contrary, orthologous relationships between papaya and spider flower/arabidopsis are relatively complex, including one-to-one, one-to-two, one-to-three, and two-to-four. In spider flower, the majority (84.6%) of duplicate pairs within an orthogroup were characterized as WGD repeats, which is relatively more than the 69.2% found in arabidopsis. Moreover, the duplication mode of the remaining duplicate pairs is also different, i.e., dispersed duplication in spider flower and tandem duplication in arabidopsis, respectively (Table S1).

Compared with other species examined, 27.5% of *AtLEA* genes seem to be arabidopsis-specific. To uncover their evolution patterns in Brassicaceae, we further traced their orthologs in representative Brassicaceae plants whose genome sequences are available in Phytozome v13, i.e., *A. lyrata*, *A. halleri*, *Capsella rubella*, *C. grandiflora*, *Eutrema salsugineum*, *Brassica oleracea*, and *B. rapa*. As expected, all of them have orthologs in at least one out of seven species examined, though species-specific evolution was observed (Table S3).

### 3.3. Exon-Intron Structure, Phylogenetic Analysis, and Structural Characterization

**Figure 3:**
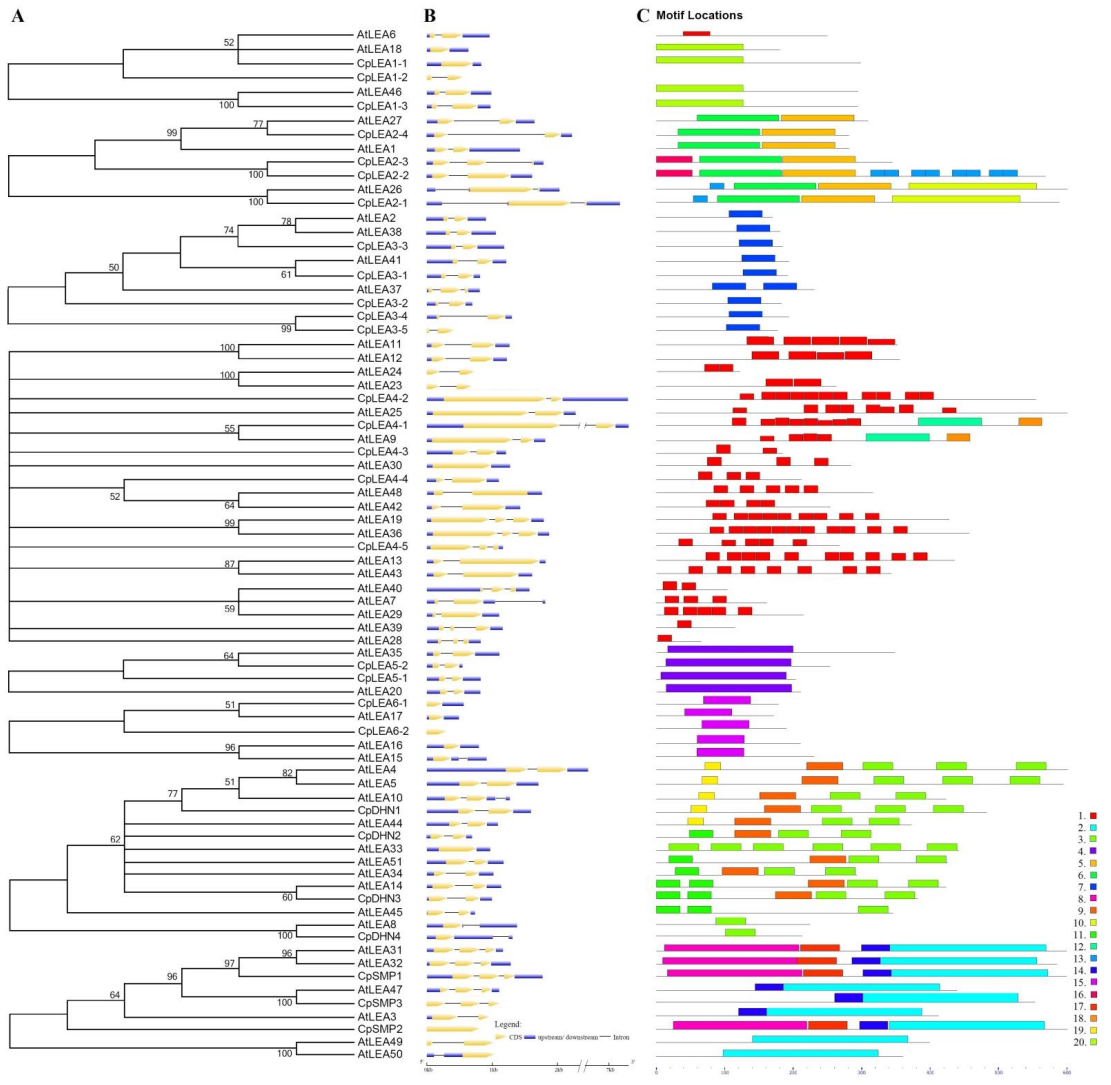
Phylogenetic analysis, gene structure, and motif distribution of papaya and arabidopsis *LEA* genes. (**A**) Phylogenetic analysis of eight families of Cp/AtLEA proteins; (**B**) Exon-intron structures of *Cp/AtLEA* genes; (**C**) Distribution of 20 conserved motifs. Multiple sequence alignments were conducted using MUSCLE and unrooted phylogenetic trees were constructed using MEGA6 (maximum likelihood method; bootstrap, 1000 replicates; shown are bootstrap values at nodes supported by a posterior probability of ≥50%). Motifs were identified using MEME.

To learn more about the divergence between papaya and arabidopsis, we performed phylogenetic analysis of LEA proteins according to families, and further compared their gene structures and protein motifs. As observed in arabidopsis, *CpLEA* genes feature few introns, varying from zero to two in the coding region, accounting for 14.3%, 75%, and 10.7% of total genes, respectively. Notably, an additional intron was also found in 5′ or 3′ untranslated regions (UTR) of *CpLEA2-1* and *CpDHN4*, though no intron is present in the coding region of *CpDHN4* (Figure 3). Moreover, 12 out of 25 intron-containing *CpLEA* genes appeared to have alternative splicing (AS) isoforms, and the proportion of 48% is relatively more than the 39.5% found in arabidopsis (Table S1). For convenience, the most expressed transcript was selected for further analyses. The deduced protein length of *CpLEA* genes varies from 78 to 590 amino acids (AA), and molecular weight (MW) and isoelectric point (pI) values range from 8.77 to 66.20 kDa, or from 4.56 to 10.07, respectively. Except for CpLEA2-4, the GRAVY value of other CpLEA proteins is less than 0, implying their hydrophilic feature. These proteins were predicted to target mitochondria, chloroplast, nuclear, cytoplasmic as well as extracellular genes (Table 1). A further MEME search resulted in 20 conserved motifs, which were shown to significantly distribute over different families (Figure 3 and Figure S2).

#### 3.3.1. LEA_1

The LEA_1 family is also known as D-113 [42]. In papaya, this family includes three members, which is equal to that of arabidopsis (Figure 2). However, their gene origin is not exactly the same. In fact, these genes belong to three phylogenetic groups or orthogroups, i.e., LEA1a, LEA1b, and LEA1c (Figure 2 and Table 2). Among them, *AtLEA18* was characterized as a paralog of *AtLEA6* that were resulted from the α WGD [43]. Whereas the majority of members in this family contain one intron, *CpLEA1-1* and *AtLEA18* in LEA1a are intronless (Figure 3), gene-specific loss of an intron can be speculated. Most proteins in this family were shown to harbor Motif 20, which was characterized as the LEA_1 domain. By contrast, despite the presence of the LEA_1 domain in CpLEA1-2 and AtLEA6 as supported by a MOTIF Search, no motif was detected in CpLEA1-2 due to the parameter of 20 motifs set in this study, whereas AtLEA6 was shown to harbor Motif 1, which was characterized as a LEA_4-like domain, supporting their sequence divergence (Figure 3). The length of three CpLEA1s varies from 102 to 160 AA, and the average of 140 AA is relatively longer than the 130 AA observed in arabidopsis. Correspondingly, the MW value varies from 11.41 to 17.01 kDa, and the average of 14.83 kDa is relatively larger than 13.85 kDa in arabidopsis (Table 1). Nevertheless, the pI value in two species appeared to be greater than 7.0, implying their basic feature.

#### 3.3.2. LEA_2

This family is also known as LEA14 or D-95 [42]. The four members found in papaya are relatively more than the three present in arabidopsis (Figure 2). Similar to LEA_1, the LEA_2 family also includes three orthogroups, i.e., LEA2a, LEA2b, and LEA2c (Table 2). In contrast to *AtLEA1* and *AtLEA27* that are repeats derived from the β WGD [43], *CpLEA2-1* was characterized as a transposed repeat of *CpLEA2-4*, which also resulted in *CpLEA2-3* via tandem duplication; and *CpLEA2-2* is a more recent tandem repeat of *CpLEA2-3* (Figure 1 and Table S1). Most genes in this family harbor a single intron in the coding region; however, *CpLEA2-3* contains two instead and the gain of the second intron can be speculated. Moreover, one more intron was also observed in the 5′ UTR of both *CpLEA2-1* and *AtLEA26*, implying their early origin. All members in this family include Motif 6 and Motif 5, which were characterized as the LEA_2 or LEA_3-like domain, respectively. Moreover, both CpLEA2-1 and AtLEA26 harbor two additional motifs, i.e., Motif 13 and Motif 10, where the latter was characterized as the LEA_2 domain; both CpLEA2-2 and CpLEA2-3 include Motif 16, while CpLEA2-2 also contains eight copies of Motif 13 (Figure 3). The length of CpLEA2s varies from 151 to 316 AA, and the average of 239 AA is relatively longer than 214 AA in arabidopsis. Correspondingly, the MW value varies from 16.16 to 35.12 kDa, and the average of 26.40 kDa is relatively larger than 23.48 kDa in arabidopsis. Nevertheless, the pI value in these two species varies from 4.53 to 5.65 (Table 1), suggesting that they are acidic.

#### 3.3.3. LEA_3

This family is also known as LEA5 or D-73 [42], and the five members present in papaya are relatively more than the four present in arabidopsis (Figure 2), which can be assigned into five orthogroups, i.e., LEA3a, LEA3b, LEA3c, LEA3d, and LEA3e (Table 1). Among them, *AtLEA38* and *AtLEA41* are repeats of *AtLEA2* and were derived from the α or γ WGD, respectively [43]; *CpLEA3-1* may also be derived from *CpLEA3-3* via the γ WGD, whereas *CpLEA3-4* was characterized as a transposed repeat of *CpLEA3-5*, which only exhibit 33.3% sequence identity at the protein level. This family features one intron; however, *AtLEA37* has gained an additional intron in the coding region. All members in this family harbor a single motif (i.e., Motif 7), which was characterized as the LEA_3 domain (Figure 3). The length of CpLEA3s varies from 95 to 104 AA, and the average of 100 AA is relatively shorter than 104 AA in arabidopsis. Correspondingly, the MW value varies from 10.61 to 11.78 kDa, and the average of 11.03 kDa is slightly smaller than 11.38 kDa in arabidopsis. The pI value in two species varies from 9.39 to 10.07 (Table 1), indicating that they are basic.

#### 3.3.4. LEA_4

This family is also known as D-7 or D-29 [42], which contains the most number of 6 or 18 members in papaya and arabidopsis, respectively (Figure 2). This family was shown to be highly diverse, including six main orthogroups and six Brassicaceae-specific groups, i.e., LEA4a, LEA4b, LEA4c, LEA4d, LEA4e, LEA4f, AtLEA7/-29, AtLEA11/-12, AtLEA23/-24, AtLEA28, AtLEA39, and AtLEA40 (Table 2 and Table S3). Among them, *AtLEA42*/*-48*, *AtLEA19*/-*36*, *AtLEA13*/-*43*, and *AtLEA7*/-*29* are duplicates that resulted from the α WGD [43], *AtLEA11*/-*12* and *AtLEA7*/-*40* are transposed repeats, and *AtLEA23*/-*24* are tandem repeats (Table S1). The intron number also varies from zero to two, and the copy number of the widely distributed Motif 1, which was characterized as the LEA_4 domain, varies from one to eleven. Additionally, both CpLEA4-1 and AtLEA9 harbor two more motifs, i.e., Motif 12 and Motif 18, where the former was characterized as a domain of unknown function (DUF4149, PF13664) (Figure 3). The length of CpLEA4s varies from 193 to 590 AA, and the average of 358 AA is considerably longer than 280 AA in arabidopsis. Correspondingly, the MW value varies from 23.63 to 61.45 kDa, and the average of 39.33 kDa is relatively smaller than 30.37 kDa in arabidopsis. Unlike most families, the pI value in both species is highly diverse, varying from 4.82 to 9.71 (Table 1).

#### 3.3.5. LEA_5

This family is also known as D-19 or EM [42], which includes two members in both papaya and arabidopsis, comprising two orthogroups, i.e., LEA5a and LEA5b (Figure 2 and Table 2). Whereas CpLEA5-1 and -2 were characterized as WGD repeats, AtLEA20 and -35 are dispersed repeats (Table S1), implying possible chromosome rearrangement after papaya-arabidopsis divergence. All members in this family feature a single intron and harbor Motif 4 that was characterized as the LEA_5 domain (Figure 3). Nevertheless, the sequence length of LEA5b is relatively longer than LEA5a (i.e., 89–92 vs. 111–152) due to fragment insertion. The MW value of CpLEA5-1 and CpLEA5-2 is 9.64 or 12.10 kDa, respectively, and the average of 10.87 kDa is relatively smaller than 13.27 kDa in arabidopsis. The pI value in two species varies from 5.51 to 6.75 (Table 1), suggesting that they are acidic.

#### 3.3.6. LEA_6

This family is also known as PvLEA18 [44], which harbors two or three members in papaya and arabidopsis, respectively (Figure 2). It is composed of two orthogroups, i.e., LEA6a and LEA6b (Table 2), where *AtLEA15* and *AtLEA16* in LEA6b are tandem repeats (Table S1). Although most genes are intronless, *AtLEA15* was shown to gain one intron in the 3′ UTR. The unique motif identified in this family (i.e., Motif 15) was characterized as the LEA_6 domain (Figure 3). CpLEA6-1 and CpLEA6-2 are 97 or 78 AA in length, respectively, and the average of 88 AA is slightly longer at 83 AA in arabidopsis, whereas the average MW value of 9.60 kDa in papaya is relatively larger than 8.71 kDa in arabidopsis. The pI value in these two species varies from 4.46 to 5.56 (Table 1), implying that they are acidic.

#### 3.3.7. DHN

This family is also known as D-11 [42], and the 4 members found in papaya is considerably less than the 10 present in arabidopsis (Figure 2). These genes constitute five orthogroups and one Brassicaceae-specific group, i.e., DHNa, DHNb, DHNc, DHNd, DHNe, and AtLEA44 (Table 2 and Table S3). Among them, *AtLEA4*/-*5* and *AtLEA33*/-*34* are tandem repeats (Table S1), where *AtLEA4*/*-10*, *AtLEA14*/-*45*, and *AtLEA33*/-*51* are duplicates that were derived from the α WGD [43]. Most members in this family harbor one intron in the coding region; however, *AtLEA33* has lost the corresponding intron present in its paralogs (i.e., *AtLEA34* and *AtLEA51*). By contrast, one conserved intron was found in the 3′ UTR of both *CpDHN4* and *AtLEA8*, though the intron retention was observed in one alternative splicing isoform of *CpDHN4*, supporting species-specific evolution. All members in this family include Motif 3, which was characterized as the DHN domain (or more precisely as the K-segment), and the motif copies vary from one to six. One copy of Motif 9, which was also characterized as the DHN domain (or more precisely as the S-segment), is widely found with the exception of CpDHN4, AtLEA8, AtLEA33, and AtLEA45. Further sequence alignment revealed the presence of the S-segment at the C-terminal of both CpDHN4 and AtLEA8, and one to three copies of the Y-segment at the N-terminal of CpDHN2, CpDHN3, AtLEA14, AtLEA34, AtLEA45, and AtLEA51. Based on the presence and order of these conserved domains, all five architectures (i.e., K_n_, SK_n_, K_n_S, Y_n_K_n_, and Y_n_SK_n_) were found in arabidopsis, while only SK_n_, K_n_S, and Y_n_SK_n_ were identified in papaya (Figure S3). Additionally, members in DHNa as well as AtLEA44 also harbor Motif 19 (Figure 3 and Figure S3), whose function has not been described yet. The length of CpDHNs varies from 93 to 211 AA, and the average of 152 AA is relatively shorter than 181 AA in arabidopsis. Correspondingly, the MW value varies from 10.50 to 24.10 kDa, and the average of 16.82 kDa is relatively smaller than 19.76 kDa in arabidopsis. Like the LEA_4 family, the pI value in both species is also diverse, varying from 4.74 to 9.38 (Table 1).

#### 3.3.8. SMP

This family is also known as D-34 [42,45], and the three members identified in papaya is considerably less than the six present in arabidopsis (Figure 2). They comprise three orthogroups and one Brassicaceae-specific group, i.e., SMPa, SMPb, SMPc, and AtLEA49/-50 (Table 2 and Table S3). Among them, *AtLEA31*/-*32* and *AtLEA49*/-*50* are tandem repeats, *AtLEA3/31* are transposed repeats, and *CpSMP2/-3* were characterized as WGD and transposed repeats of *CpSMP1*, respectively. Members in SMPa and SMPc feature two introns, whereas other group members have no or a single one instead. Despite the close evolutionary relationship between *AtLEA49* and *AtLEA50*, they include one intron in the coding region or 5′ UTR, respectively, implying fast evolution and sequence divergence. All members in this family include Motif 2, which was characterized as the SMP domain. Moreover, Motif 14 is also present in members of SMPa, SMPb, and SMPc, whereas two more motifs (i.e., Motif 8 and Motif 17) were also found in members of SMPa and SMPb (Figure 3). Noteworthy, Motif 8 was also characterized as the SMP domain, implying possible fragment duplication or gene fusion. The length of CpSMPs varies from 244 to 267 AA, and the average of 258 AA is relatively longer than 204 AA in arabidopsis. Correspondingly, the MW value varies from 25.13 to 27.97 kDa, and the average of 26.60 kDa is relatively larger than 21.15 kDa in arabidopsis. The pI value in the two species varies from 4.56 to 6.44 (Table 1), indicating that they are acidic.

### 3.4. ABRE and LTRE cis-Acting Elements Present in the Promoter Region of CpLEA Genes

**Figure 4:**
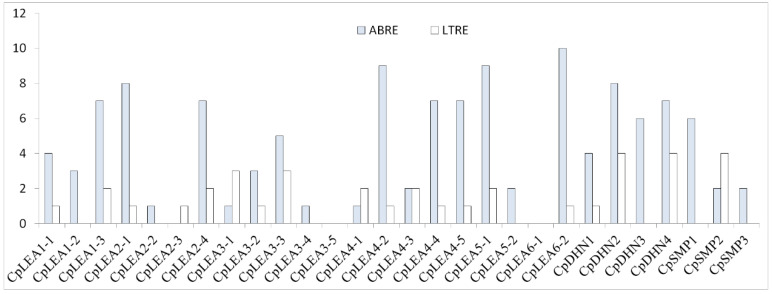
ABRE and LTRE *cis*-acting elements present in 2000-bp promoter regions of *CpLEA* genes.

LTRE, also known as DRE (drought responsive) or CRT (C-repeat), is a key *cis*-acting element for CBF/DREB1 transcription factors, whereas ABRE is a key element involved in ABA signaling [46,47]. Previous studies showed that these two elements are overrepresented in the promoter region of *AtLEA* genes and are associated with ABA, cold and/or drought responses [3]. To reveal possible response patterns of *CpLEA* genes to stresses, we examined the presence of ABRE and LTRE elements in the 2,000-bp promoter regions. Results showed that 89.3% of *CpLEA* genes contain 1 to 10 copies of the ABRE element, only excluding *CpLEA2-3*, *CpLEA3-5*, and *CpLEA6-1*, while 67.9% of them contain 1 to 4 copies of the LTRE element, excluding *CpLEA1-2*, *CpLEA2-2*, *CpLEA3-4*, *CpLEA3-5*, *CpLEA5-2*, *CpLEA6-1*, *CpDHN3*, *CpSMP1*, and *CpSMP3* (Figure 4). The proportion is similar to the 82.0% and 69.0% reported for *AtLEA* genes, respectively [3].

### 3.5. Tissue-Specific Expression Profiles of CpLEA Genes

Although some LEA proteins have been reported to be regulated by posttranslational modifications (e.g., phosphorylation), cellular trafficking, homo- and heteromerization [18,48,49,50], and transcriptional regulation still represent a key mechanism to perform their functions. For this purpose, we first performed global expression profiling of *CpLEA* genes in various tissues.

**Figure 5:**
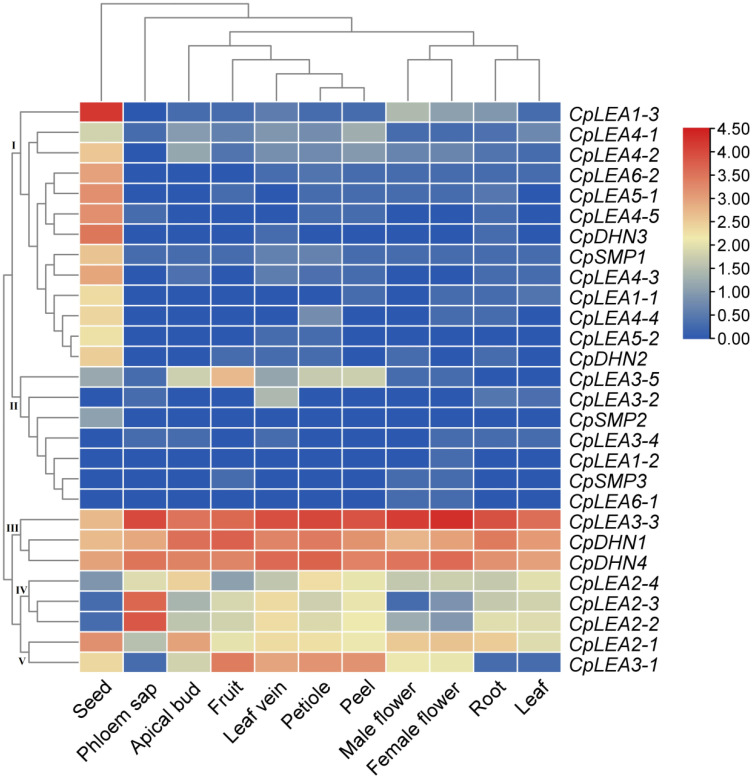
Tissue-specific expression profiles of *CpLEA* genes. Color scale represents FKPM normalized log_10_ transformed counts, where blue indicates low expression and red indicates high expression.

As shown in Figure 5, our transcriptional profiling supported the expression of all *CpLEA* genes in at least one of 11 tissues examined in this study, i.e., root, apical bud, leaf, petiole, leaf vein, phloem sap, male flower, female flower, fruit, peel, and seed, though the transcript level was highly diverse. As expected, *CpLEA* genes were most expressed in the seed, but considerably less expressed in the leaf and root, which is consistent with the cluster analysis. In total, 22 out of 28 *CpLEA* genes (75.9%) possessed a FKPM value >1 in the seed, which is relatively more than the 15 in the petiole, 15 in the vein, 13 in the root, 12 in the bud, 11 in the fruit, 11 in the peel, 10 in the leaf, 10 in the female flower, 9 in the male flower, and 7 in the sap. Five genes, i.e., *CpLEA3-3*, *CpDHN4*, *CpDHN1*, *CpLEA2-1*, and *CpLEA2-4*, appeared to constitutively express in these tissues, whereas other genes were tissue-specific. As for a certain tissues, several key genes were also identified: *CpLEA3-3* represents the most expressed gene in most tested tissues, whereas *CpLEA1-3* and *CpDHN1* represent the most expressed genes in the seed or bud/fruit, respectively; *CpDHN4* represents the second most expressed gene in the male flower, female flower, petiole, vein, and peel, whereas *CpLEA3-3*, *CpDHN1*, *CpDHN3*, and *CpLEA2-2* represent the second most expressed genes in the bud/fruit, root/leaf, seed, or sap, respectively. According to tissue-specific expression patterns, *CpLEA* genes can be divided into five main clusters: Cluster I includes the most of the 13 genes that are predominantly expressed in the seed; Cluster II includes *CpLEA3-5* (preferentially expressed in fruit), *CpLEA3-2* (preferentially expressed in vein), *CpSMP2* (preferentially expressed in seed), and other four rarely expressed genes; Cluster III includes *CpLEA3-3*, *CpDHN1*, and *CpDHN4*, which are constitutively expressed; Cluster IV includes *CpLEA2-2*, *CpLEA2-3*, and *CpLEA2-4*, which are typically expressed in sap; and Cluster V includes the constitutively expressed *CpLEA2-1*, as well as *CpLEA3-1*, which is preferentially expressed in fruit (Figure 5).

### 3.6. Expression Patterns of CpLEA Genes during Fruit Development

**Figure 6:**
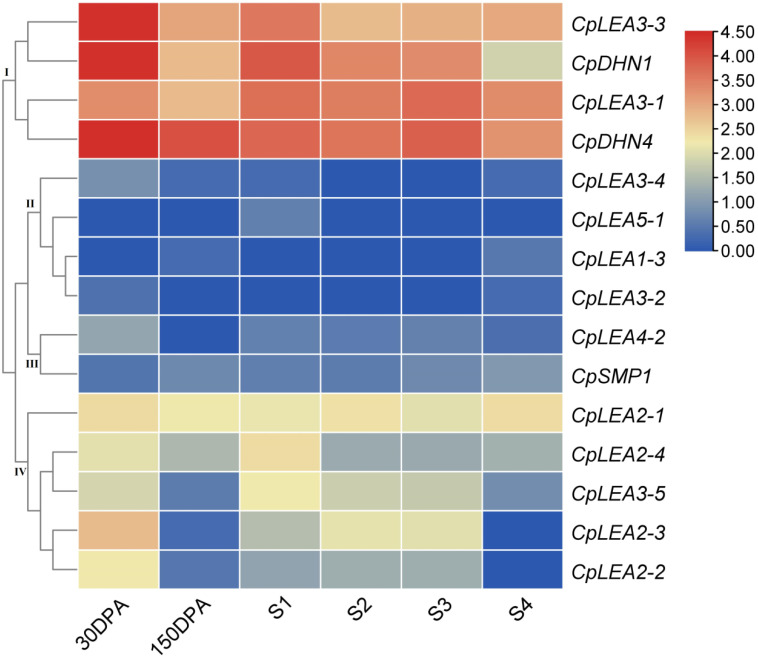
Expression profiles of *CpLEA* genes during fruit development. Color scale represents FKPM normalized log_10_ transformed counts, where blue indicates low expression and red indicates high expression. (DPA, days post-anthesis; S, stage of developmental fruit).

To learn more about the expression pattern of *CpLEA* genes during fruit development, six typical stages were investigated, i.e., 30 days post-anthesis (30 DPA), 150 DPA, and stages 1–4 of fruit flesh from immature to ripe, i.e., S1, S2, S3, and S4, as previously defined [51]. Unlike rapid accumulation of *LEA* genes during the late stage of seed development as described in other species, *CpLEA* genes were shown to be the most expressed in the early stages of fruit development, but considerably less expressed in mature fresh fruit. Based on the expression patterns of 15 genes with the FKPM value >1 in at least one of the stages tested, these genes could be grouped into four clusters: Cluster I includes *CpLEA3-1*, *CpLEA3-3*, *CpDHN1*, and *CpDHN4*, which were highly abundant in all stages; Cluster II includes *CpLEA1-3*, *CpLEA3-2*, *CpLEA3-4*, and *CpLEA5-1*, which were rarely or lowly expressed in a few stages; Cluster III includes *CpLEA4-2* and *CpSMP1*, which were lowly expressed in most stages; Cluster IV includes *CpLEA2-1*, *CpLEA2-2*, *CpLEA2-3*, *CpLEA2-4*, and *CpLEA3-5*, which were moderately expressed in most stages (Figure 6).

### 3.7. Expression Patterns of CpLEA Genes under Drought, Cold and Salt Stresses

**Figure 7:**
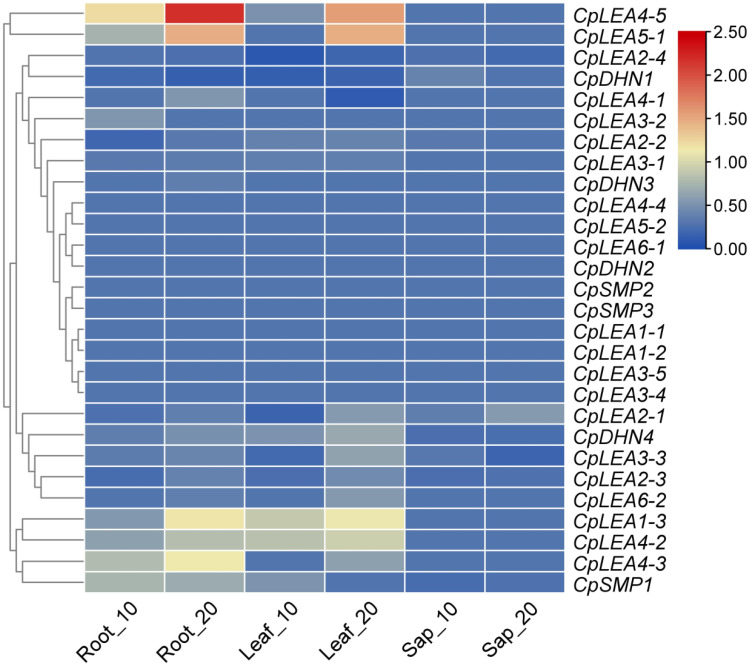
Expression profiles of *CpLEA* genes upon drought stress. The FKPM value of all genes in controls was normalized to one, and the color scale represents normalized log_10_ transformed fold changes, where blue indicates low expression and red indicates high expression.

The response of *CpLEA* genes to mild (10 d) and severe (20 d) drought was investigated based on transcriptomes of the roots, leaves, and phloem sap [36]. As shown in Figure 7, a total of 15 *CpLEA* genes were differentially expressed in at least one tissue per treatment, and the majority of them (86.7%) were shown to be significantly up-regulated. As for the root, six genes, i.e., *CpLEA1-3*, *CpLEA4-2*, *CpLEA4-3*, *CpLEA4-5*, *CpLEA5-1*, and *CpSMP1*, were up-regulated under both conditions; *CpLEA3-2* was up-regulated by mild drought, whereas *CpLEA4-1* and *CpDHN4* were up-regulated by severe drought; by contrast, *CpDHN1* was down-regulated by severe drought. As for the leaf, in contrast to the down-regulation of *CpDHN1*, four genes, i.e., *CpLEA1-3*, *CpLEA4-2*, *CpLEA4-5*, and *CpDHN4*, were up-regulated by both treatments; *CpSMP1* was up-regulated only by mild drought, whereas *CpLEA2-1*, *CpLEA3-3*, *CpLEA4-3*, *CpLEA5-1*, and *CpLEA6-2* were up-regulated only by severe drought; *CpLEA2-4* and *CpLEA4-1* were down-regulated by mild and severe drought, respectively. As for the sap, only one gene (i.e., *CpLEA2-1*) was up-regulated by severe drought (Figure 7).

**Figure 8:**
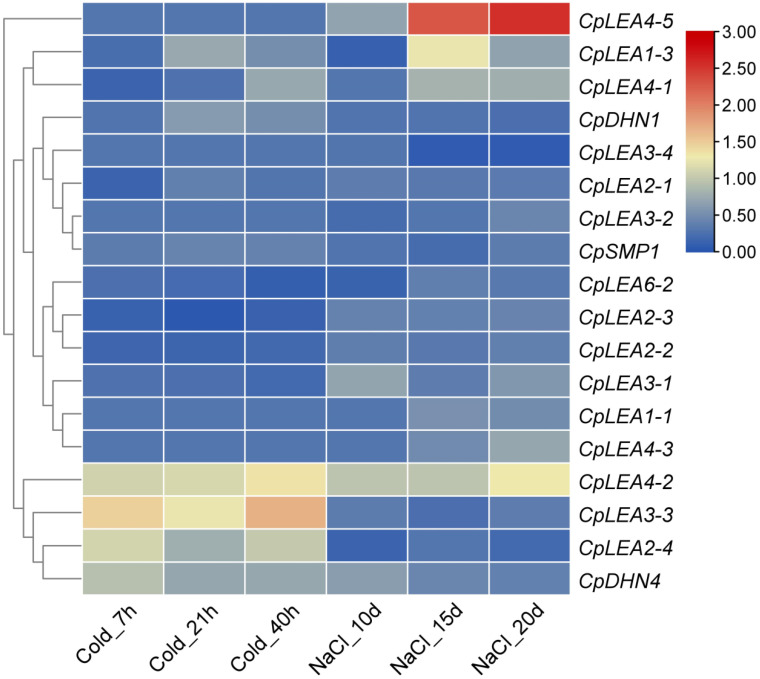
Expression profiles of *CpLEA* genes upon cold and salt stresses. The FKPM value of all genes in the controls was normalized to one, and the color scale represents normalized log_10_ transformed fold changes, where blue indicates low expression and red indicates high expression.

To study the response of *CpLEA* genes to cold and salt stresses, eight-week-old plantlets were subjected to 4 °C chilling or 300 mM/L NaCl treatment, and the leaf transcriptome was characterized at 0–40 h or 0–20 d post treatment, respectively. Among 18 *CpLEA* genes with a FKPM value >1, 16 genes were shown to be significantly regulated: six (i.e., *CpLEA1-3*, *CpLEA2-4*, *CpLEA4-1*, *CpLEA4-2*, *CpLEA6-2*, and *CpDHN4*) are shared by cold and salt stresses, whereas five (i.e., *CpLEA2-1*, *CpLEA2-2*, *CpLEA2-3*, *CpLEA3-3*, and *CpDHN1*) and five (i.e., *CpLEA1-1*, *CpLEA3-1*, *CpLEA3-4*, *CpLEA4-3*, and *CpLEA4-5*) are cold- or salt-specific, respectively. Similar to drought stress, most genes were up-regulated, accounting for about 68.8% of total *LEA* genes, though some of them (i.e., *CpLEA1-3*, *CpLEA2-4*, and *CpLEA4-1*) were occasionally down-regulated at a certain time point. As for cold stress, five regulated genes are shared by three time points, including four up-regulated (i.e., *CpLEA2-4*, *CpLEA3-3*, *CpLEA4-2*, and *CpDHN4*) and one down-regulated (i.e., *CpLEA2-3*); *CpLEA2-2* was down-regulated at two former time points, whereas *CpLEA1-3* and *CpDHN1* were up-regulated at the latter two time points; *CpLEA2-1* and *CpLEA6-2* were down-regulated at 7 or 40 h post-treatment, respectively; *CpLEA4-1* was down-regulated at 7 h but up-regulated at 40 h post-treatment. As for salt stress, *CpLEA4-2* and *CpLEA4-5* were up-regulated at three time points, whereas *CpLEA1-3* was down-regulated at 10 d but up-regulated at the latter two time points; *CpLEA1-1*, *CpLEA4-1*, and *CpLEA4-3* were up-regulated at the latter two time points, whereas *CpLEA3-4* was down-regulated at the same time points; *CpDHN4* was up-regulated at 10 d post-treatment, whereas *CpLEA2-4* and *CpLEA6-2* were down-regulated at the same time point; *CpLEA3-1* was up-regulated at 10 and 20 d post-treatment (Figure 8).

## 4. Discussion

### 4.1. Small Number but High Diversity of LEA Genes in Papaya

Although first identified for their accumulation in the later stages of seed development, LEA proteins have been found in a wide range of plant tissues, as well as different types of organisms [1,7,21,45]. In contrast to a single or few members present in algae, rapid expansion of the *LEA* superfamily was observed in terrestrial plants, which was shown to be essential for survival under water stress [9,52]. Rapid gene expansion is usually accompanied by WGDs, which are widespread and play an important role in the radiation of flowering plants [53]. In eudicots, studies established that the γ whole genome triplication event occurred at 117 million years ago (Mya), sometime before the diversification of core eudicots [54]. After that, arabidopsis, a Brassicaceae plant within the order Brassicales, was proven to experience two additional whole genome doubling events, i.e., β and α, occurred within a window of 61–65 and 23–50 Mya, respectively [19,55]. As a result, a high number of 51 *LEA* genes are present in arabidopsis, including seven dispersed repeats as well as 21 repeats that resulted from γ WGD (1), β WGD (1), α WGD (9), tandem duplication (7), and transposed duplication (4) (Table S1).

In this study, a first genome-wide identification of *LEA* genes was conducted in an important tropical fruit tree of the Caricaceae family, papaya, as well as another two Brassicales plants, i.e., horseradish tree and spider flower. Horseradish tree is an important multipurpose shrub with medicinal and nutritional properties and the ability to grow in the low water conditions of the Moringaceae family, whereas spider flower belongs to a phylogenetic outgroup of the Brassicaceae sister family Cleomaceae [21,22]. Like castor bean (Euphorbiaceae), the papaya and horseradish tree did not experience any additional WGD after the γ WGD. By contrast, the spider flower shared the β WGD but further experienced one genome triplication that is independent of the Brassicaceae-specific α WGD as described in arabidopsis [19,20,21,22,56]. As expected, a relatively small number of 28 or 29 *LEA* genes were found in the papaya and horseradish tree, respectively, which are comparable to 28 reported in castor bean, but relatively less than the 39 and 51 present in spider flower and arabidopsis, respectively, reflecting the occurrence of lineage-specific WGDs in the latter after their divergence [3,8,19,21].

*LEA* genes identified in this study belong to eight out of nine families as described in arabidopsis, i.e., LEA_1, LEA_2, LEA_3, LEA_4, LEA_5, LEA_6, DHN, and SMP [3]. As for the AtM family, which includes two tandem repeats in arabidopsis, it is more likely to be Brassicaceae-specific, because it is widely present in Brassicaceae plants (Table S3) but has not yet been identified in other species [3,8,9,12,13], including species examined in this study. Nevertheless, 28 *CpLEA* genes represent 27 out of 29 orthogroups based on sequence comparison of the above five species, though a LEA4f homolog has lost the corresponding LEA_4 domain. Moreover, no orthologs were identified for CpLEA1-2, CpLEA2-2, CpLEA2-3, CpLEA3-4, or CpLEA3-5 in arabidopsis, though their counterparts are present in at least one of three other species examined.

### 4.2. Comparative Genomics Analysis Reveals Lineage-Specific Evolution of the LEA Superfamily in Brassicales

Orthology defines genes in different organisms that evolved from a common ancestral gene via speciation, which may perform similar functions [57]. Characterization of 29 orthogroups in five representative species allows us to infer lineage-specific evolution in Brassicales. Notably, a nearly one-to-one orthologous relationship was observed between the papaya/horseradish tree and castor bean, though they belong to different plant families, implying that few *LEA* genes have been lost in either the papaya or horseradish tree after the split with the castor bean. By contrast, tandem duplication plays a predominant role in gene expansion within an orthogroup, i.e., *RcDHN2/-3* in DHNb, and *RcSMP1/-2* in SMPb, *CpLEA2-2/-3* in LEA2b, and *MoLEA5-2/-3* in LEA5b. As for the spider flower, which experienced two WGDs (including the β WGD shared by Brassicaceae plants) after the split with papaya at approximately 72 Mya [21,58], duplicate pairs are mainly contributed by WGD (12), followed by dispersed duplication (3) and transposed duplication (1) (Table S1). The transposed duplication is shared by all five species examined, whereas WGD repeats appear to be spider flower-specific. By contrast, *AtLEA2/-41* and *AtLEA1/-27* were characterized as γ and β WGD-derived repeats, respectively [22], supporting species-specific evolution following WGDs. Nevertheless, since the spider flower-specific WGD is a triplication event, theoretically, it should have given rise to three gene copies from a single ancestral gene. However, in most cases, only one or two copies are maintained. Unlike the spider flower, tandem duplication also plays a key role in gene expansion in arabidopsis.

Further comparative analysis of exon-intron structures and protein motifs revealed frequent gain and/or loss of certain introns/motifs, which includes the loss of the second intron in *CpLEA2-3* relative to *CpLEA2-2*. In fact, compared with papaya, such an occurrence is relatively more prevalent in arabidopsis, which is consistent with a relatively faster evolution of annual than perennial shrubs [59]. Nevertheless, family-specific Pfam domains are highly conserved. It is worth noting that CpLEA2-1 and AtLEA26 contain two LEA_2 domains relative to a single one present in other LEA_2 family members, implying a possible fragment repetition. From an evolutionary perspective, further characterization of these species-specific genes is of particular interest.

### 4.3. Diverse Expression Patterns of CpLEA Genes and a Role in Fruit Development and Abiotic Stress Responses

As reported in other species, our transcriptional profiling revealed diverse expression patterns of *CpLEA* genes in 11 tissues, as well as six typical stages of fruit development examined in this study. In contrast to the constitutive expression of a few members, e.g., *CpLEA2-1*, *CpLEA2-4*, *CpLEA3-3*, *CpDHN1*, and *CpDHN4*, most *CpLEA* genes appeared to preferentially express in a few tissues, especially in seed. However, except for *CpLEA1-3* and *CpSMP1* that preferentially accumulated in mature fruits, the expression patterns of most *CpLEA* genes differ from that observed in seeds, which undergo a dehydration process [2,3,4,5,6,21]. The high abundance of *CpDHN4*, *CpDHN1*, *CpLEA3-1*, and *CpLEA3-3* in fruits implies their possible important role in this special tissue.

Analyzing promoter sequences of *CpLEA* genes revealed the presence of a high number of ABRE and LTRE *cis*-acting elements, implying their possible involvement in stress responses. As expected, the transcript levels of most *CpLEA* were shown to be significantly regulated by the cold, drought, and high salt conditions examined in this study. Among three genes (i.e., *CpLEA2-3*, *CpLEA3-5*, and *CpLEA6-1*) without ABRE elements in their promoters, none of them were regulated by drought as well as salt, though *CpLEA2-3* was down-regulated by cold, which is consistent with the presence of one copy of the LTRE element in its promoter. Among nine genes (i.e., *CpLEA1-2*, *CpLEA2-2*, *CpLEA3-4*, *CpLEA3-5*, *CpLEA5-2*, *CpLEA6-1*, *CpDHN3*, *CpSMP1*, and *CpSMP3*) without LTRE elements, only *CpLEA2-2* was shown to be down-regulated by cold, while *CpLEA3-4* and *CpSMP1* were regulated by salt or drought, respectively. Among 20 genes containing both ABRE and LTRE *cis*-acting elements, most of them (85.0%) were regulated by at least one of the three stresses tested, only excluding *CpLEA4-4*, *CpDHN2*, and *CpSMP2*, which were preferentially expressed in seed but lowly expressed in the leaf, root and sap examined in this study. Among these 17 regulated genes, all of them were up-regulated by at least one treatment in at least one of three examined tissues: nine genes (i.e., *CpLEA1-1*, *CpLEA3-1*, *CpLEA3-2*, *CpLEA3-3*, *CpLEA4-2*, *CpLEA4-3*, *CpLEA4-5*, *CpLEA5-1*, and *CpDHN4*) exhibit a single up-regulated pattern; *CpLEA2-4*, the unique gene regulated in sap, was up-regulated by drought but down-regulated by cold in leaf; *CpDHN1*, a cold-induced gene, was down-regulated by drought in both the root and leaf; *CpLEA2-4* was up-regulated by cold but down-regulated by both drought and NaCl in the leaf; *CpLEA6-2* was down-regulated by both cold and NaCl but up-regulated by drought in the leaf; *CpLEA4-5*, a NaCl-induced gene, was down-regulated in leaf but up-regulated in root upon drought stress; by contrast, an initial decline followed by a steady increasing trend was observed. Regulation by stresses has been frequently reported in arabidopsis, rice, cassava (*Manihot esculenta*), and other species [2,3,12,13]. In arabidopsis, a study revealed that 54.5% of genes highly expressed in non-seed tissues were induced more than threefold by various stresses, mainly by cold, drought and salt [3]. For example, *AtLEA18*, the ortholog of *CpLEA1-1*, was also induced by salt; *AtLEA41*, the ortholog of *CpLEA3-1*, was induced by ABA, cold, and salt; *AtLEA46*, the ortholog of *CpLEA1-3*, was induced by ABA, cold, drought, and salt [2,3]. Thereby, similar functions could be speculated.

## 5. Conclusions

This study presents the first genome-wide identification of *LEA* genes in papaya as well another two Brassicales plants, horseradish tree and spider flower; resulting in 28, 29, and 39 members, respectively. These genes belong to eight out of nine families as described in arabidopsis, i.e., LEA_1, LEA_2, LEA_3, LEA_4, LEA_5, LEA_6, DHN, and SMP. Further comparison of *LEA* genes in papaya, horseradish tree, spider flower, castor bean, and arabidopsis reveals lineage-specific evolution in Brassicales, and significant expansion in spider flower and arabidopsis was mainly contributed by WGDs sometime after their split with papaya. Analysis of exon-intron structures and protein motifs supported the fast evolution of this special family, especially in arabidopsis. Moreover, global expression profiles of *CpLEA* genes were comprehensively analyzed, which revealed tissue-specific expression patterns and key roles in fruit development and stress responses. Taken together, these findings provide valuable information for further functional analysis of *LEA* genes in papaya and other species.

## Figures and Tables

**Table 1:** *LEA* genes identified in papaya.

Family	Gene Name	Locus		AS	Deduced Protein				
		Sunset	ASGPBv0.4		AA	MW (kDa)	pI	GRAVY	Loc
LEA_1	*CpLEA1-1*	sunset05G0006380	evm.TU.supercontig_18.65	-	160	17.01	9.65	−0.755	Nucl
	*CpLEA1-2*	sunset05G0013060	evm.TU.supercontig_41.41	-	102	11.41	7.03	−0.908	Mito
	*CpLEA1-3*	sunset08G0019430	evm.TU.supercontig_85.72	Yes	158	16.07	8.83	−0.878	Mito
LEA_2	*CpLEA2-1*	sunset05G0003590	evm.TU.supercontig_9.242	Yes	316	35.12	4.69	−0.384	Cyto
	*CpLEA2-2*	sunset05G0009060	evm.TU.supercontig_11.66	Yes	305	34.10	5.38	−0.243	Chlo
	*CpLEA2-3*	sunset05G0009070	evm.TU.supercontig_11.68	Yes	185	20.23	5.65	−0.056	Chlo
	*CpLEA2-4*	sunset05G0009080	evm.TU.supercontig_11.69	Yes	151	16.16	4.75	0.094	Cyto
LEA_3	*CpLEA3-1*	sunset03G0023320	evm.TU.supercontig_16.192	-	103	11.21	10.07	−0.472	Chlo
	*CpLEA3-2*	sunset04G0017920	evm.TU.supercontig_25.184	Yes	98	10.94	9.52	−0.526	Chlo
	*CpLEA3-3*	sunset05G0003680	evm.TU.supercontig_9.251	Yes	99	10.61	9.89	−0.531	Cyto
	*CpLEA3-4*	sunset05G0018090	evm.TU.supercontig_2471.1	Yes	95	10.62	9.66	−0.997	Mito
	*CpLEA3-5*	sunset06G0002130	evm.TU.supercontig_200.7	-	104	11.78	9.69	−0.839	Cyto
LEA_4	*CpLEA4-1*	sunset01G0016400	evm.TU.supercontig_66.6	-	590	66.20	8.91	−0.515	Extr
	*CpLEA4-2*	sunset03G0025310	evm.TU.supercontig_209.19	-	581	61.45	5.20	−0.864	Nucl
	*CpLEA4-3*	sunset05G0000220	evm.TU.supercontig_146.20	-	193	21.63	5.21	−1.053	Extr
	*CpLEA4-4*	sunset07G0004690	evm.TU.supercontig_464.2	-	222	24.57	8.95	−1.333	Chlo
	*CpLEA4-5*	sunset08G0016230	evm.TU.supercontig_5.110	Yes	280	30.34	6.17	−1.360	Nucl
LEA_5	*CpLEA5-1*	sunset02G0011780	evm.TU.supercontig_19.160	-	89	9.64	5.51	−1.319	Cyto
	*CpLEA5-2*	sunset08G0009640	evm.TU.supercontig_2485.2	-	111	12.10	5.51	−1.338	Nucl
LEA_6	*CpLEA6-1*	sunset01G0017510	evm.TU.supercontig_88.61	-	97	10.42	5.56	−0.705	Nucl
	*CpLEA6-2*	sunset04G0003310	evm.TU.supercontig_6.54	-	78	8.77	5.22	−1.573	Nucl
DHN	*CpDHN1*	sunset01G0014930	evm.TU.supercontig_26.225	Yes	211	24.10	5.05	−1.584	Nucl
	*CpDHN2*	sunset04G0004410	evm.TU.supercontig_6.176	-	137	14.76	9.45	−1.222	Nucl
	*CpDHN3*	sunset06G0003520	evm.TU.supercontig_106.3	Yes	167	17.93	5.94	−1.265	Nucl
	*CpDHN4*	sunset06G0021280	evm.TU.supercontig_161.14	Yes	93	10.50	6.62	−1.984	Nucl
SMP	*CpSMP1*	sunset03G0005590	evm.TU.supercontig_58.99	-	262	26.70	4.70	−0.270	Chlo
	*CpSMP2*	sunset03G0027120	evm.TU.supercontig_487.3	-	267	27.97	4.56	−0.246	Cyto
	*CpSMP3*	sunset06G0024460	evm.TU.contig_34050.2	-	244	25.13	6.44	−0.359	Nucl

AA, Amino acid; AS, Alternative splicing; Chlo, Chloroplast; Cyto, Cytoplasmic; Extr, Extracellular; GRAVY, Grand average of hydropathicity; Mito, Mitochondria; MW, Molecular weight; Nucl, Nuclear; pI, Isoelectric point; Loc, Subcellular localization.

**Table 2:** 29 Orthogroups identified in this study.

Family	Orthogroup	Papaya	Horseradish Tree	Spider Flower	Castor Been	Arabidopsis
LEA_1	LEA1a	CpLEA1-1	MoLEA1-1	ThLEA1-1 ThLEA1-2	RcLEA1-2	AtLEA6 AtLEA18
	LEA1b	CpLEA1-2	MoLEA1-2	-	RcLEA1-1	-
	LEA1c	CpLEA1-3	MoLEA1-3	ThLEA1-3 ThLEA1-4	RcLEA1-3	AtLEA46
LEA_2	LEA2a	CpLEA2-1	MoLEA2-1	ThLEA2-1 ThLEA2-2	RcLEA2-2	AtLEA26
	LEA2b	CpLEA2-2 CpLEA2-3	-	ThLEA2-3 ThLEA2-4 ThLEA2-5 ThLEA2-6	-	-
	LEA2c	CpLEA2-4	MoLEA2-2	ThLEA2-7	RcLEA2-1	AtLEA1 AtLEA27
LEA_3	LEA3a	CpLEA3-1	MoLEA3-1	ThLEA3-1	RcLEA3-5	AtLEA41
	LEA3b	CpLEA3-2	MoLEA3-2	ThLEA3-2	RcLEA3-4	AtLEA37
	LEA3c	CpLEA3-3	MoLEA3-3	ThLEA3-3 ThLEA3-4	RcLEA3-1	AtLEA2 AtLEA38
	LEA3d	CpLEA3-4	MoLEA3-4	ThLEA3-5	RcLEA3-2	-
	LEA3e	CpLEA3-5	MoLEA3-5	-	RcLEA3-3	-
LEA_4	LEA4a	CpLEA4-1	MoLEA4-1	ThLEA4-1	-	AtLEA9
	LEA4b	CpLEA4-2	MoLEA4-2	ThLEA4-2	RcLEA4-2	AtLEA25
	LEA4c	CpLEA4-3	MoLEA4-3	-	RcLEA4-4	AtLEA30
	LEA4d	CpLEA4-4	MoLEA4-4	ThLEA4-3	RcLEA4-3	AtLEA42 AtLEA48
	LEA4e	CpLEA4-5	MoLEA4-5	ThLEA4-4	RcLEA4-5	AtLEA19 AtLEA36
	LEA4f	-	MoLEA4-6	ThLEA4-5 ThLEA4-6	RcLEA4-1	AtLEA13 AtLEA43
LEA_5	LEA5a	CpLEA5-1	MoLEA5-1	ThLEA5-1	RcLEA5-1	AtLEA20
	LEA5b	CpLEA5-2	MoLEA5-2 MoLEA5-3	ThLEA5-2	RcLEA5-2	AtLEA35
LEA_6	LEA6a	CpLEA6-1	MoLEA6-1	ThLEA6-1	RcLEA6-1	AtLEA17
	LEA6b	CpLEA6-2	MoLEA6-2	ThLEA6-2	RcLEA6-2	AtLEA15 AtLEA16
DHN	DHNa	CpDHN1	MoDHN1	ThDHN1 ThDHN2	RcDHN1	AtLEA4 AtLEA5 AtLEA10
	DHNb	CpDHN2	MoDHN2	ThDHN3	RcDHN2 RcDHN3	AtLEA33 AtLEA34 AtLEA51
	DHNc	CpDHN3	MoDHN3	ThDHN4 ThDHN5	RcDHN4	AtLEA14 AtLEA45
	DHNd	CpDHN4	MoDHN4	ThDHN6 ThDHN7 ThDHN8	RcDHN5	AtLEA8
	DHNe	-	MoDHN5	ThDHN9	-	-
SMP	SMPa	CpSMP1	MoSMP1	ThSMP1	RcSMP3	AtLEA31 AtLEA32
	SMPb	CpSMP2	MoSMP2	ThSMP2	RcSMP1 RcSMP2	AtLEA3
	SMPc	CpSMP3	MoSMP3	ThSMP3 ThSMP4	RcSMP4	AtLEA47

## Supplementary Materials

The following supporting information can be downloaded at: https://www.mdpi.com/article/10.3390/life12091453/s1, Figure S1: Nucleotide and protein sequence alignments of *CpLEA2-2* and *CpLEA2-3*; Figure S2: Sequence logos of 20 motifs identified in this study; Figure S3: Alignment of DHN proteins in papaya and arabidopsis; Table S1: Detailed information of *LEA* genes present in papaya, horseradish tree, spider flower, castor bean, and arabidopsis; Table S2: Detailed information of transcriptome data used in this study; Table S3: Orthologs in representative Brassicaceae plants for 14 arabidopsis-specific *LEA* genes identified in this study.
