# Supplementary figures and images for: Genomic Analysis of LEA Genes in Carica papaya and Insight into Lineage-Specific Family Evolution in Brassicales
Source: Life (Basel). 2022 Sep 19;12(9):1453. doi: 10.3390/life12091453 (PMC9502557; doi:10.3390/life12091453)

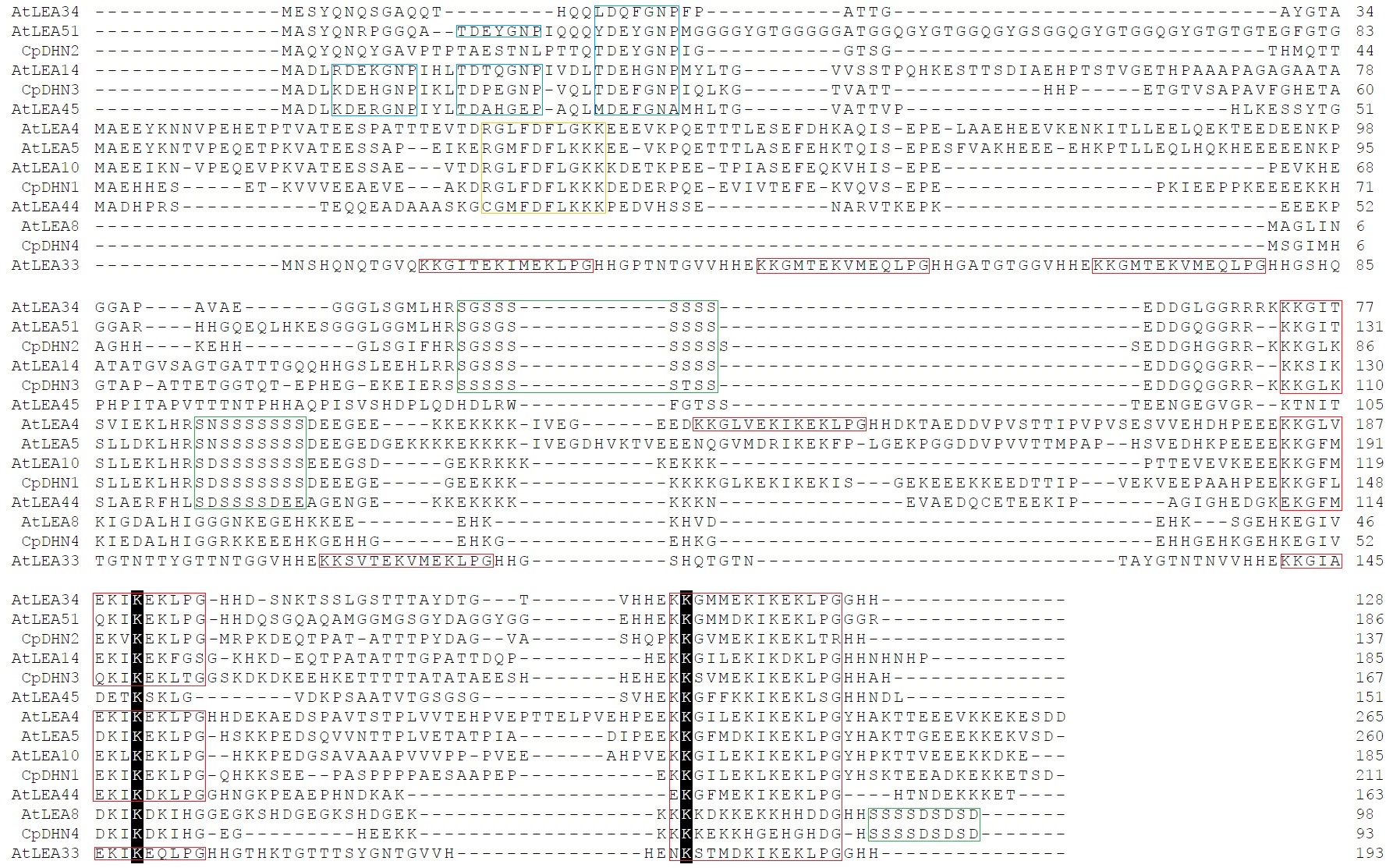

Supplement: Supplementary file 1 [file life-12-01453-s001.zip › Figure S3.jpg]

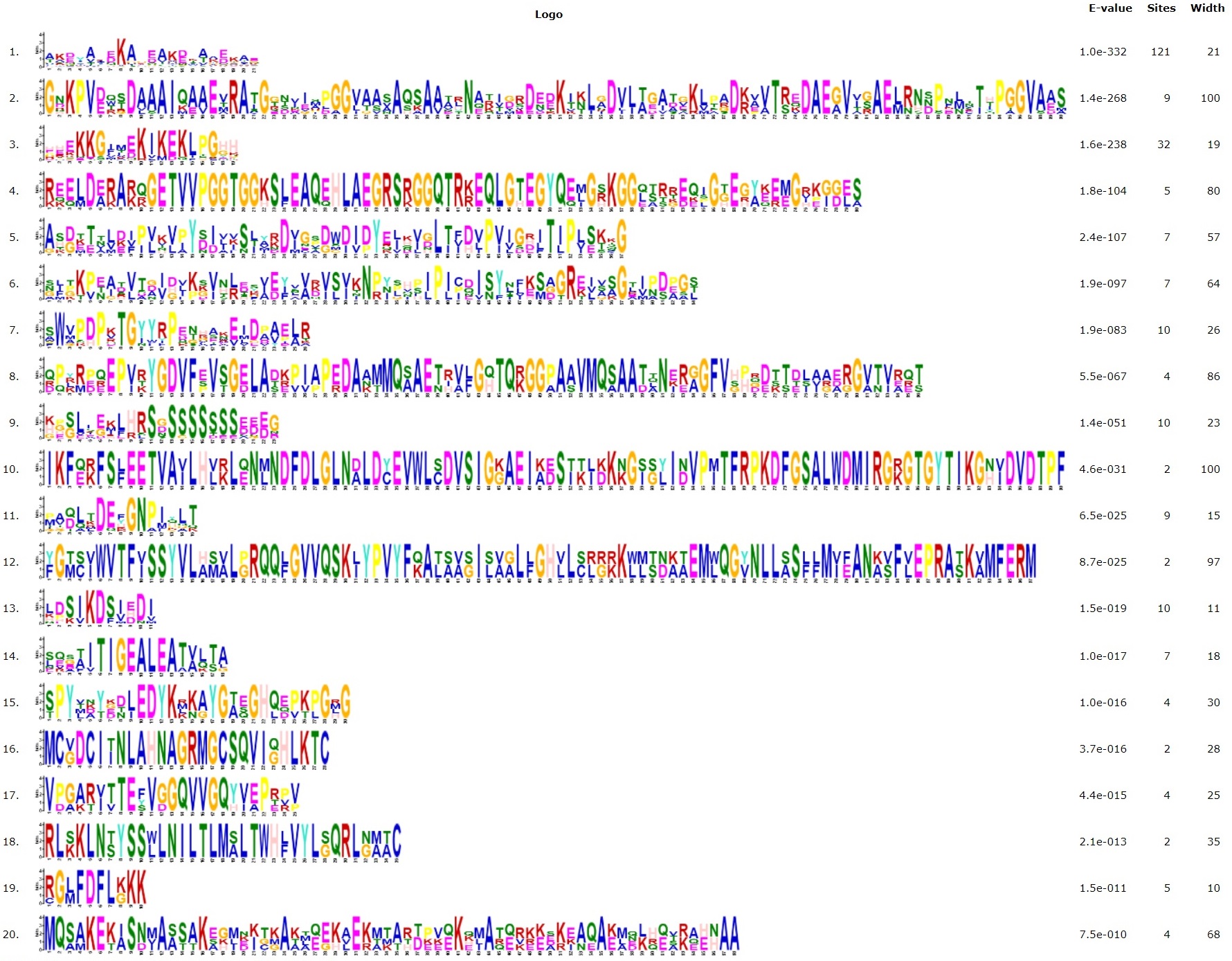

Supplement: Supplementary file 1 [file life-12-01453-s001.zip › Figure S2.jpg]

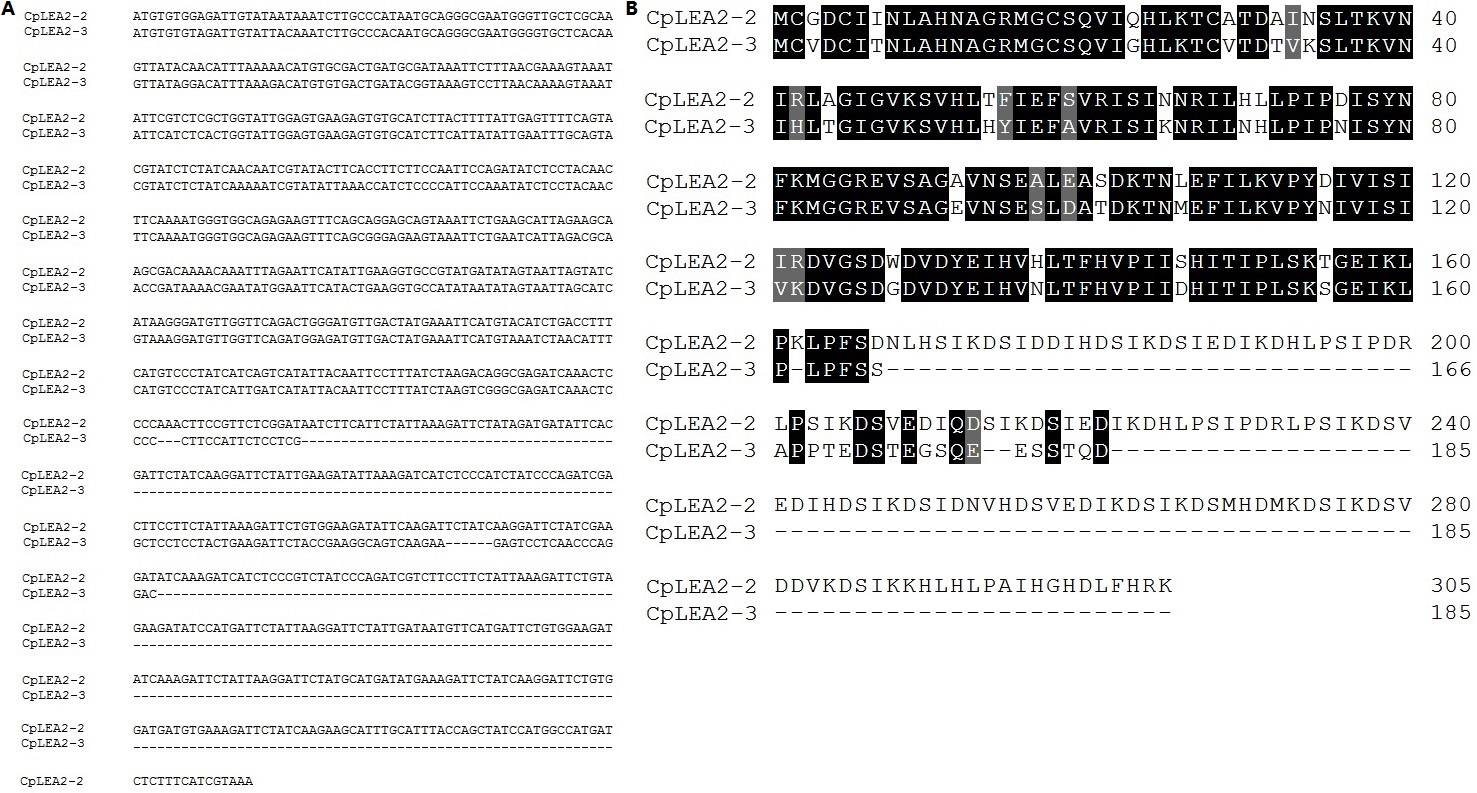

Supplement: Supplementary file 1 [file life-12-01453-s001.zip › Figure S1.jpg]
